# A case of endosalpingiosis in the lymph nodes of the mesocolon

**DOI:** 10.1186/s40792-020-00942-6

**Published:** 2020-07-23

**Authors:** Koichiro Niwa, Kazuhiro Sakamoto, Michitoshi Goto, Yutaka Kojima, Makoto Takahashi, Shun Ishiyama, Masaya Kawai, Yuu Okazawa, Natumi Tomita, Eiichiro Seki, Yuki Fukumura, Takashi Yao

**Affiliations:** 1 Department of Coloproctological Surgery, Juntendo University Faculty of Medicine, 2-1-1 Hongo, Bunkyo-ku, Tokyo, 113-8421 Japan; 2 Department of Gastroenterological Surgery, Juntendo University Faculty of Medicine, 2-1-1 Hongo, Bunkyo-ku, Tokyo, 113-8421 Japan; 3 Department of Surgery, Ohta General Hospital, 1-50 Nisshin-cho, Kawasaki-ku, Kanagawa 210-0024 Japan; 4 Department of Human Pathology, Juntendo University Faculty of Medicine, 2-1-1 Hongo, Bunkyo-ku, Tokyo, 113-8421 Japan

**Keywords:** Endosalpingiosis, Endometriosis, Laparoscopic surgery

## Abstract

**Background:**

Endosalpingiosis in the lymph nodes of the mesocolon is very rare. We reported a case with appendiceal endometriosis who had endosalpingiosis in the lymph nodes of the mesocolon that was found during laparoscopic ileocecal resection.

**Case presentation:**

The patient was a 44-year-old woman who had visited a physician for fever, bloody stool, and abdominal pain 1 year earlier. She was diagnosed with ulcerative colitis on colonoscopy, and symptoms improved with oral treatment. A colonoscopy performed 2 months after diagnosis detected a hard, 20-mm submucosal tumor (SMT) in the cecum. On abdominal contrast CT, an intensely stained mass, including a low-density region, was observed in the cecum. A boring biopsy was performed after mucosal resection of the cecal SMT at our hospital, but diagnosis could not be made. Since the possibility of a malignant lesion could not be ruled out, laparoscopic ileocecal resection was performed. In the resected specimen, a 29 × 27 × 21-mm mass was present in the appendicular root. On histopathological examination, appendiceal endometriosis and endosalpingiosis in the lymph nodes around the ileocolic artery were observed. The postoperative course was favorable, and the patient was discharged 7 days after surgery.

**Conclusion:**

Differentiation of endosalpingiosis in lymph nodes in the mesocolon from lymph node metastasis of adenocarcinoma is important in patients with an abdominal mass.

## Background

Endosalpingiosis is considered as ectopic benign epithelial lesion histologically similar to tubal epithelium, and many lesions are observed in either the peritoneum, great omentum, urinary bladder, or pelvic and para-aortic lymph nodes [1–3]. When endosalpingiosis is present in a lymph node, it is important to differentiate it from lymph node metastasis of adenocarcinoma [4, 5]. We encountered a patient who underwent laparoscopic ileocecal resection for a diagnosis of cecal tumor in which endosalpingiosis was noted in the lymph nodes in the mesocolon with appendiceal endometriosis. We report the case with a literature review.

## Case presentation

The patient was a 44-year-old woman who visited a physician for fever, bloody stool, and abdominal pain 1 year earlier. She was diagnosed with ulcerative colitis on colonoscopy, and symptoms improved with oral treatment. A colonoscopy performed 2 months after diagnosis detected a hard, 20-mm submucosal tumor (SMT) in the cecum, and the patient was referred to our hospital for additional examination.

Her past medical history included polyostotic fibrous dysplasia that was followed for 2 years and ulcerative colitis treated orally for 1 year. There was no particular familial medical history, and her pregnancy/delivery history was 2 gravida 1 para.

The findings of her first examination by us were as follows: height, 164 cm; body weight, 41 kg; body temperature, 36.5 °C; blood pressure, 102/64 mmHg; and pulse, 84/min. No jaundice or anemia was noted in the palpebral conjunctiva. The superficial lymph nodes were not palpable.

Her blood chemistry showed no abnormal finding other than a mild increase in a tumor marker, CEA (≦ 3.0 ng/dl), to 3.4 ng/dl.

**Fig. 1:**
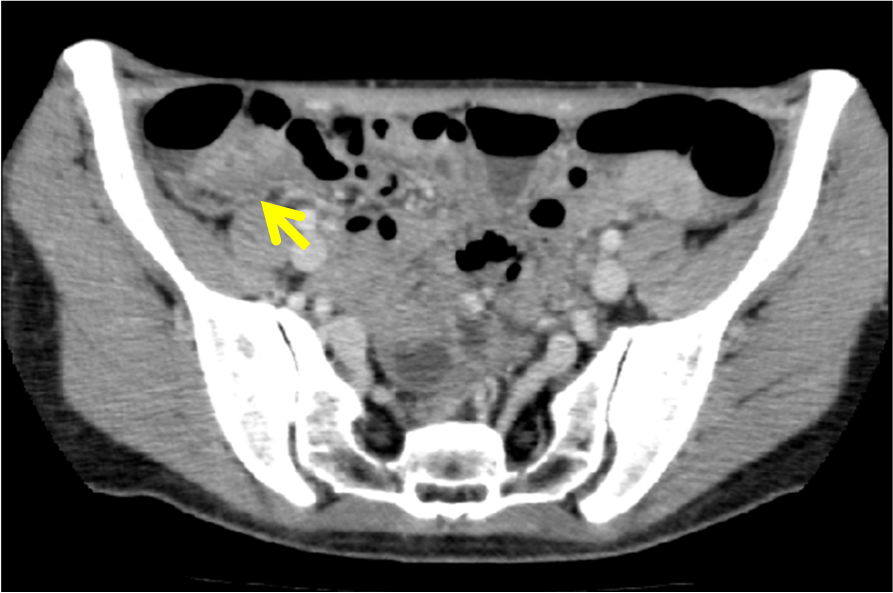
On abdominal CT, an intensely stained mass with a 30-mm diameter, including a low-density region (arrow), was observed in the cecum

On abdominal CT, an intensely stained mass with a 30-mm diameter, including a low-density region, was observed in the cecum. No lymph node swelling was noted around either the cecum or ileocolic artery (Fig. 1).

**Fig. 2:**
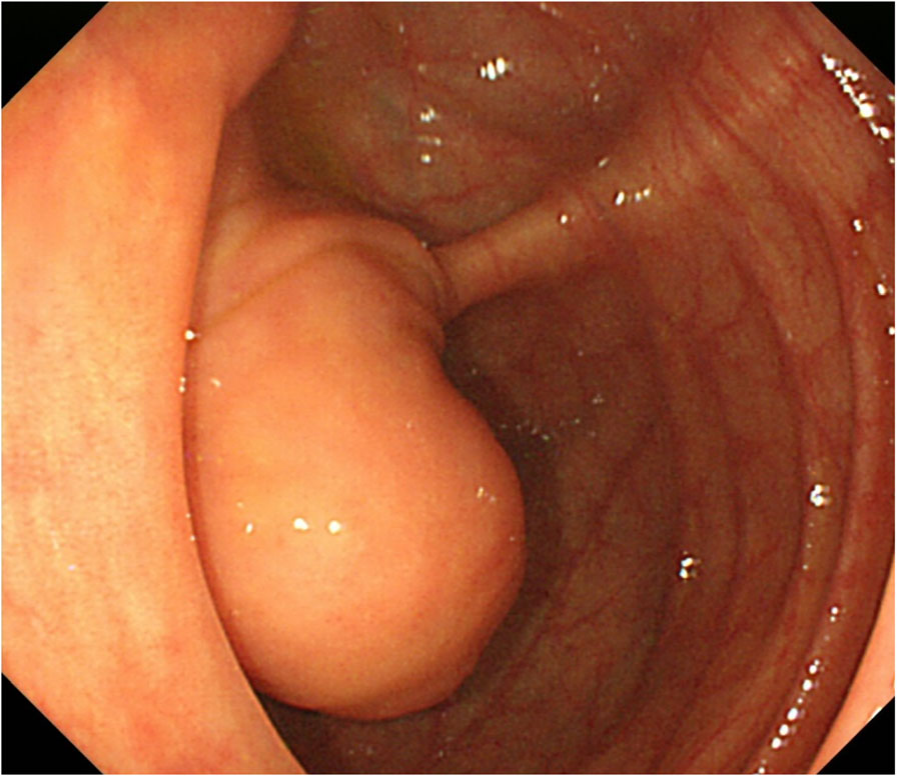
SMT with a 20-mm diameter was noted in the cecum on colonoscopy

SMT with a 20-mm diameter was noted in the cecum on colonoscopy. A boring biopsy was performed after resection of the mucosa using a snare, but a tumorous lesion was not observed (Fig. 2).

**Fig. 3:**
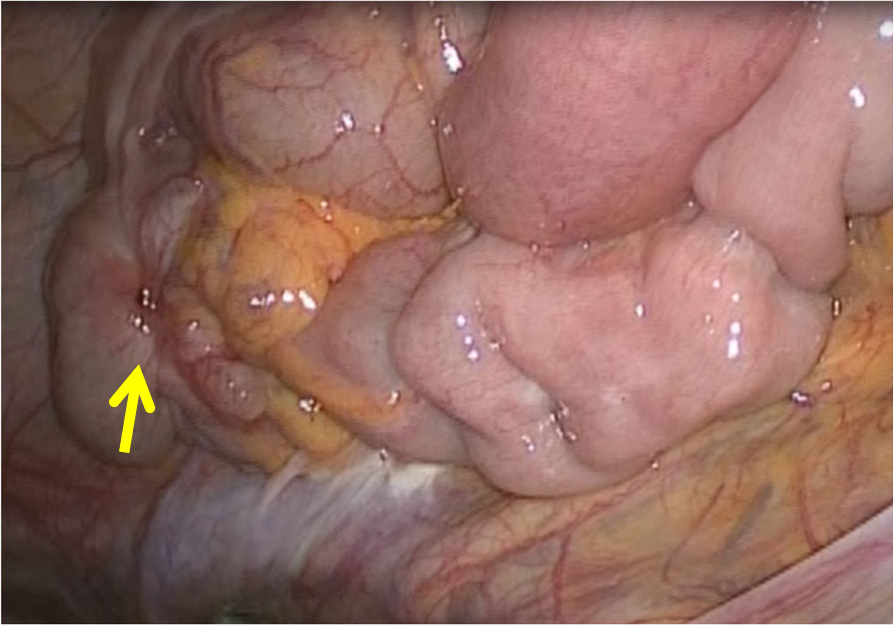
The appendix could not be confirmed, and twitching and hardening of the serosa (arrow) were observed in the ileocecal region

Laparoscopic surgery was performed for a diagnosis of the cecal tumor. The appendix could not be confirmed, and twitching and hardening of the serosa were observed in the ileocecal region, for which laparoscopic ileocecal resection was performed following the procedure for malignant disease. Lymph nodes were dissected up to the origin of the ileocolic artery following D3 dissection. No obviously enlarged lymph node was evident during surgery (Fig. 3).

**Fig. 4:**
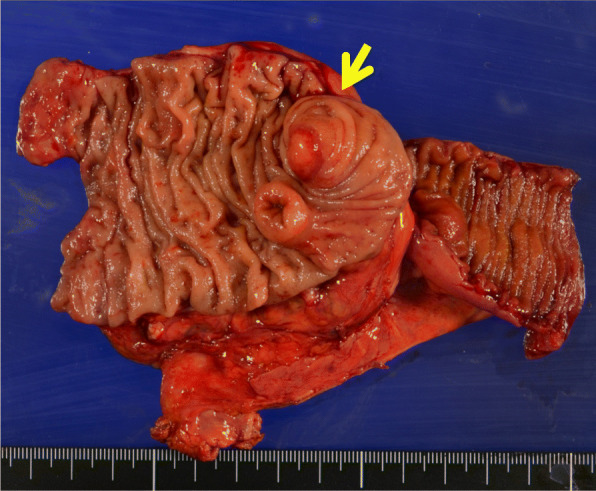
The excised specimen contained a submucosal tumor-like protruding lesion (arrow) at a location consistent with the appendiceal orifice

The excised specimen contained a submucosal tumor-like protruding lesion at a location consistent with the appendiceal orifice. The appendix was very short and showed marked deformity (Fig. 4).

**Fig. 5:**
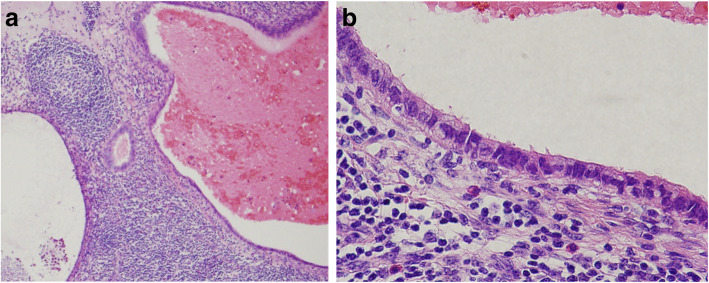
**a** Endometrial glands were present in lymph nodes around the ileocolic artery, and there were only small changes in the gland structure (H&E, low magnification). **b** Luminal structures accompanied by pilus structures were present in the lymph nodes around the ileocolic artery (H&E, high magnification)

On histopathological examination, high columnar epithelium accompanied by endometrial stroma was observed in the ileum over the cecum, mainly in the appendiceal muscular layer and subserosa. This finding provided the rationale for the diagnosis of appendiceal endometriosis. Twenty-three lymph nodes around the ileocolic artery were excised. Two of them were covered with a columnar epithelial monolayer and showed luminal structures accompanied by pilus structures, and these were diagnosed as endosalpingiosis of lymph nodes in the mesocolon (Fig. 5).

The postoperative course was favorable. The patient started oral ingestion 2 days after surgery and was discharged at 7 days after the surgery. The tumor marker level (CEA) was maintained at about 1–2 ng/dL after surgery.

## Discussion

Endosalpingiosis was initially used in 1930 by Sampson to describe the tubal epithelial lesions observed in scar tissue after salpingectomy [6], and it was considered due to implantation after salpingectomy. However, it is now defined as the ectopic presence of benign epithelium histologically similar to tubal epithelium [7]. The most frequent development site is the peritoneum, and it is also observed in the omentum, urinary bladder, and pelvic and para-aortic lymph nodes [8]. Endosalpingiosis is observed in 5.3% of the pelvic lymph nodes dissected to treat gynecological malignant tumors [9]. In our case, endosalpinx tissue was present in the lymph nodes around the ileocolic artery in the mesocolon, and it was diagnosed as endosalpingiosis of the lymph nodes. Endosalpingiosis of the lymph nodes in the mesocolon is a very rare case [10]. There are various hypotheses for the development of endosalpingiosis, such as implantation, metaplasia, transport, and aberrant metaplasia. Transport and aberrant metaplasia are considered potential hypotheses for endosalpingiosis of lymph nodes, but it is still unclear [9]. Since endosalpingiosis was not present in tissue other than in the lymph nodes around the ileocolic artery in our patient, the transport and aberrant metaplasia hypotheses were considered. There are case reports on malignancy [11], but the details, such as the frequency and mechanism, are not defined.

Unlike endometriosis, subjective symptoms are absent in many cases of endosalpingiosis because neither the growth or inflammation is consistent with the menstrual cycle. Fewer cases are reported by surgeons and gynecologists compared with the number of reports on endometriosis. Endosalpingiosis and endometriosis can be distinguished by normal H&E staining. The histological characteristic of endosalpingiosis is the presence of ciliated cells, non-ciliated pale secretory cells containing pale cytoplasm, and peg-shaped intercalated cells in which the cytoplasm, including the nucleus, is mostly present in the upper cellular region in gland ducts [1–3]. In many studies, however, endosalpingiosis may have been confused with endometriosis because awareness of endosalpingiosis was low. It has recently been recognized by surgeons and gynecologists as a separate disease, and it is believed that there will be an increase in case reports.

In our patient, the level of the tumor marker CEA was mildly elevated before surgery. Regarding endosalpingiosis, there has been no report indicating any association with CEA and any association between endometriosis and CEA is negative [12]. The course of CEA was followed because of the possibility of other malignant diseases, but the CEA level decreased, suggesting that the preoperative elevation was a false positive.

Since endosalpingiosis is recognized as a 1–2-mm gray cystic or papillary lesion, its differentiation from a disseminated malignant tumor and metastasis is difficult. It is also difficult to differentiate it from metastasis of malignant tumor when it is present in a lymph node, as histological investigation is necessary. Dallenbach-Hellweg summarized and reported points of differentiation between malignant tumor and endosalpingiosis [13].

Including our patient, cases with endosalpingiosis in mesentery lymph nodes are often complicated by intestinal endometriosis [10], suggesting an association with intestinal endometriosis. Surgeons may encounter patients with intestinal endometrium in whom a mass for which malignance cannot be ruled out and intestinal resection must be performed because of intestinal obstruction. In that case, recognition of the presence of endosalpingiosis may increase with reports of endosalpingiosis and enable investigation of the developmental mechanism and transformation to malignancy.

## Conclusions

We treated a patient with endosalpingiosis in the lymph nodes in the mesocolon. At present, many points remain unclear with regard to the details of endosalpingiosis. It is expected that surgeons will increasingly recognize endosalpingiosis and increase the number of case reports, which will enable close further investigation.

## Data Availability

All data generated or analyzed during this study are included in this published article.

## Abbreviations

SMT: Submucosal tumor
